# Femtosecond modulation of electron correlations in a Luttinger liquid

**DOI:** 10.1126/sciadv.aec7494

**Published:** 2026-05-27

**Authors:** Na Li, Umang Mehta, Matin Lebrat, Shunye Gao, Tika Kafle, Richa Sapkota, Henry C. Kapteyn, Oscar Granas, Rahul Nandkishore, Margaret M. Murnane

**Affiliations:** ^1^ Department of Physics and JILA, University of Colorado and NIST, Boulder, CO 80309, USA.; ^2^ Department of Physics and Center for Theory of Quantum Matter, University of Colorado Boulder, Boulder, CO 80309, USA.; ^3^ Center for Integrated Nanotechnologies, Los Alamos National Laboratory, Los Alamos, NM 87545, USA.; ^4^ KMLabs Inc., 4775 Walnut Street, #102, Boulder, CO 80301, USA.; ^5^ Division of Materials Theory, Department of Physics and Astronomy, Uppsala University, Box-516, Uppsala, SE 75120, Sweden.

## Abstract

Luttinger liquids emerge in one-dimensional metals with strong electron interactions, exhibiting intriguing near-equilibrium properties such as spin-charge separation and power-law correlations. Although these interactions suggest fast, distinctive out-of-equilibrium dynamics, such phenomena remain largely unexplored on ultrashort timescales. Here, we use femtosecond laser excitation to weakly deplete the electron density in the Luttinger band of Li_0.9_Mo_6_O_17_ and track the response via time- and angle-resolved photoemission spectroscopy. By fitting the measured electron distributions to a finite-temperature Luttinger liquid model, we observe a fast drop in the Luttinger exponent, quantifying the strength of electron interactions. Subsequently, unlike hot electrons in conventional Fermi liquids that slowly relax within picoseconds via electron-phonon coupling, hot electrons in Li_0.9_Mo_6_O_17_ relax within a short time of ~100 femtoseconds, through the excitation of a nonequilibrium collective plasmon. The extremely fast evolution of the Luttinger exponent and electron temperature—including a tens of femtosecond time lag between excitation, recovery, and plasmon-driven modulation—reveals previously unidentified pathways for modulating quantum many-body interactions in low-dimensional materials.

## Teaser

Electrons in a 1D quantum metal can cool 10 times faster than in simple metals by exciting a plasmon rather than phonons.

## INTRODUCTION

Low-dimensional materials may enable faster, lightweight, energy-efficient technologies using their emerging properties such as high-temperature superconductivity or the tailorable nature of their electronic, magnetic, and photonic properties (*1*, *2*). In particular, one-dimensional (1D) electronic systems exhibit distinct quantum phenomena compared to higher-dimensional material systems, due to the presence of enhanced electron-electron correlations and reduced screening (*3*, *4*). In such systems, conventional Fermi liquid (FL) theory breaks down. A well-known type of non-FL behavior called a Luttinger liquid (LL) can emerge, characterized by collective excitations and spin-charge separation (*5*). The strength of electron-electron correlations can be quantified by the LL anomalous exponent α (*3*, *5*) describing the power-law scaling of the electron density of states (DOS) close to the Fermi level. A conventional metal such as TiTe_2_ or Bi_2_Sr_2_CaCu_2_O_8_ that is well described by FL theory, if fit to an LL distribution, would correspond to an α close to zero (*6*, *7*). As the strength of the electron-electron interactions increases, α also increases (*8*).

While the static properties of a few well-characterized LLs have been investigated—most notably carbon nanotubes (*9*, *10*) and molybdenum bronze (*7*, *11*–*13*)—it remains largely unknown how these non-Fermi states respond to external perturbations on ultrafast timescales, how strong interactions and collective excitations dictate the material response, or whether the LL state remains stable under external perturbation. Prior studies have shown that the electronic behavior in those LL materials can be tuned via equilibrium means such as stoichiometric variations (*14*), applied pressure (*15*), and thermal expansion (*16*), which modify the dimensional crossover or the emergence of ordered states, as well as interchain coupling due to lattice parameter changes. However, whether the LL state and its associated excitations persist under nonequilibrium excitation has yet to be experimentally established.

To investigate the ultrafast response of LLs, a well-characterized prototypical LL system is optimal. Among the few quasi-1D solids (*9*, *17*–*19*) thought to exhibit LL behavior, Li_0.9_Mo_6_O_17_ (LMO) stands out because its LL nature has been validated by numerous static studies. Band structure calculations (*13*, *20*) along with static angle-resolved photoemission spectroscopy (ARPES) measurements (*3*, *11*, *21*–*25*) have demonstrated hallmark LL properties, including a linear band dispersion and spin-charge separation, with characteristic low-energy holon and spinon excitations near the Fermi level *E*_F_ (*6*, *21*, *22*). Moreover, both ARPES and tunneling spectroscopy measurements consistently show a power-law suppression of the DOS near *E*_F_, with α values between ∼0.8 at 300 K and 0.6 to 0.75 at low temperatures (*11*, *24*, *25*)*.*

**Fig. 1.**
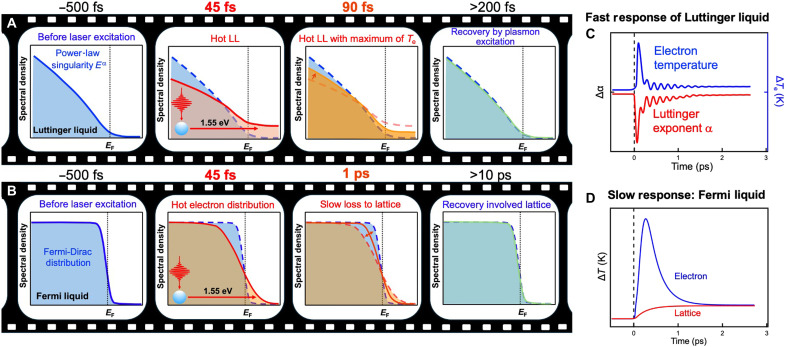
Comparison of the quasiparticle distributions and their responses in an LL and an FL. (**A**) In an LL, laser-excited electrons relax very rapidly to a hot Luttinger distribution, followed by fast cooling to the ground state within 200 fs via strong electron-electron interactions and excitation of a nonequilibrium collective plasmon. (**B**) In an FL, laser-excited electrons lose energy to the lattice via electron-phonon coupling on comparatively slow picosecond timescales. (**C**) Representative fast transient response in an LL, showing the ultrafast evolution of the electron temperature and the Luttinger exponent α on femtosecond timescales. (**D**) Representative slow transient response in an FL, illustrating evolution of the electron and lattice temperature occurring on picosecond timescales.

Here we use femtosecond laser excitation to weakly deplete the electron density in the Luttinger band of LMO. We then track the resulting electronic response via time- and ARPES. A carefully optimized low pump fluence was used to preserve the coherence of the LL band and avoid entering a regime where the LL spectral function becomes distorted. By fitting the experimentally measured electron distributions to a finite-temperature LL model, we extract the instantaneous Luttinger exponent α and electron temperature. This reveals a fast drop of ∼14% in the Luttinger exponent α within the 45-fs laser pulse, corresponding to a reduction in the many-body electronic interactions. Subsequently, unlike hot electrons in conventional FLs that slowly relax within picoseconds due to electron-phonon coupling, hot electrons in LMO relax within a short timescale of ∼100 fs via strong electron-electron interactions (see Fig. 1). The fast modulation of the electron density then launches oscillations of both the Luttinger exponent and the electron temperature, with a frequency of ∼6.6 THz and a relative phase lag of a few tens of femtoseconds. We attribute these oscillations to the excitation of a nonequilibrium collective plasmon that modulates the electronic band structure, with a delayed response of the electronic distribution due to band filling (see Fig. 1). Together, these findings establish that electron correlations in a 1D LL can be altered on femtosecond timescales, revealing previously unidentified pathways for modulating quantum many-body interactions in low-dimensional materials.

## RESULTS

**Fig. 2.**
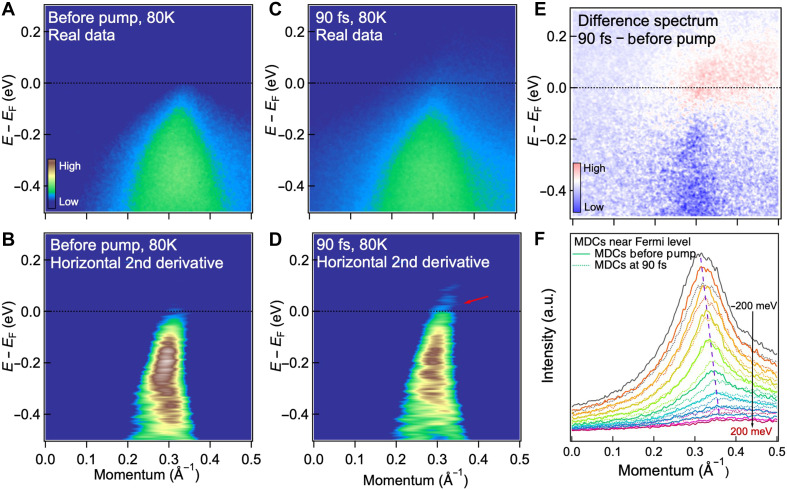
Comparison of ARPES band structures before and after the laser pump pulse for LMO, at an initial temperature of 80 K. (**A** and **B**) Raw ARPES spectra and their horizontal second derivatives before the pump. (**C** and **D**) Corresponding spectra at 90 fs after a pump pulse with a fluence of 0.5 mJ/cm^2^. Excited electrons are highlighted by the red arrow in (D). (**E**) Difference spectrum between the raw spectra at 90 fs and before the pump, showing excited electrons above the Fermi level and a depletion of spectral weight in the Luttinger band. The spectral weight above *E*_F_ appears broadened—not confined to a single band—suggesting contributions from other higher-lying 3D bands into which electrons are excited from lower 3D bands. This multiband excitation scenario is supported by the calculated band structure, where dispersive 3D bands (green) lie just above the Luttinger band (red) at *E*_F_ (see fig. S3). (**F**) Momentum distribution curves (MDCs) near the Fermi level (−0.2 to 0.2 eV). Solid lines represent data before the pump, and dotted lines show the 90-fs results. The nearly linear band dispersion is visible in both cases guided by the dashed line. a.u., arbitrary units.

The quasi-1D nature of LMO is characterized by an electronic band with linear dispersion, as demonstrated previously (*11*, *25*) and as shown in the experimental ARPES spectra shown in Fig. 2, measured at an initial sample temperature of 80 K. This is further corroborated by first-principles calculations, showing a Luttinger band with linear dispersion of 12.1 eV**·**Å for an energy range of more than 0.2 eV above and below the Fermi level, as seen in fig. S3A. Upon photoexcitation by a laser pump pulse (1.6-eV photon energy, 45-fs duration, and fluence of 0.5 mJ/cm^2^), the electron density in the Luttinger band is depleted, while excited states above the Fermi level are populated (Fig. 2, C to E). Most of the electron density relaxes back into the original band within 90 fs of the initial change, as shown in Fig. 2E. Throughout this process, the Luttinger band preserves its linear dispersion with minimal renormalization, as confirmed by fits to the momentum distribution curves in Fig. 2F. This robustness indicates that, under the low-fluence excitation used here, the LL character of the band remains intact. This is in contrast to the dimensional crossover reported for strong perturbation under equilibrium conditions (*15*, *16*), where the coupling between LMO chains is enhanced and their quasi-1D nature is destroyed. Analyzing first-principles results based on time-dependent density functional theory, we estimate that any renormalization is on the order of the thermal broadening; see fig. S3 (A and B).

**Fig. 3.**
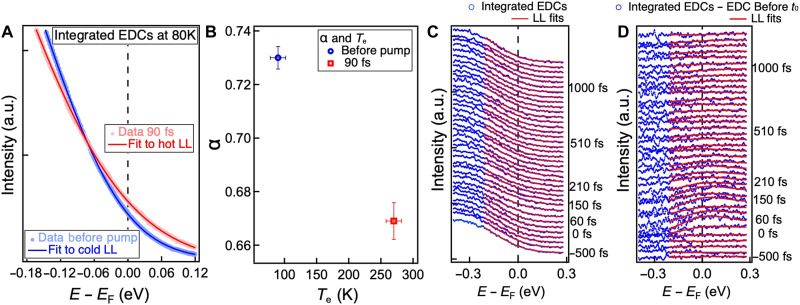
Integrated EDCs and LL model fits near *E*_F_, before and after the laser pump pulse. (**A**) Comparison of integrated ARPES EDCs and corresponding LL model fits described by Eq. 1, before and 90 fs after the laser pump pulse. Solid lines represent the fits to the experimental data shown in dots. (**B**) Extracted LL exponent α and electron temperature *T*_e_ from the fits in (A), with typical fitting uncertainties of ±0.01. Only a unique set of α and *T*_e_ provides a good fit, as detailed in section S2. (**C**) LL model fits (red lines) to integrated EDCs (blue circles) at various time delays. The LL model yields consistently good agreement with the data across all time delays. (**D**) Integrated EDCs before and after laser excitation. Significant changes can be seen from 60 to 200 fs.

We compare the DOS ρ from the ARPES spectrum through momentum integrated energy density curves near *E*_F_. As shown in Fig. 3A, the DOS (blue), although crossing the Fermi level, exhibits a strong power-law suppression of spectral weight near *E*_F_, characteristic of an LL. After laser excitation, the lineshape (red) exhibits reduced curvature near *E*_F_ and enhanced spectral weight above it. In contrast to thermal broadening observed in static temperature-dependent measurements (*11*, *25*), the changes in the Luttinger band slope are due to a rapid redistribution of electrons after photoexcitation, while the lattice remains at the base temperature of 80 K.

To quantify the changes in the LL distribution, which are captured effectively by the Luttinger exponent α and the electronic temperature *T*_e_, we extract the DOS near *E*_F_ and fit it to a power-law finite-temperature LL model

$$\rho \left(\varepsilon ,\alpha ,T_{e}\right)\propto T_{e}^{\alpha }\text{Re}\left[{\left(2i\right)}^{\alpha +1}\;B\left(\frac{\alpha +1-i\varepsilon /\pi }{2},-\alpha \right)\right]$$ (1)

where ε = (*E* − *E*_F)_/*k*_B_*T*_e_ is the scaled electron energy, *k*_B_ is the Boltzmann constant, and *B* is the beta function (*26*). At equilibrium, we extract high values of α, ∼0.73 ± 0.01 at 80 K, consistent with previous static ARPES studies (Fig. 3, A and B) (*11*). Within 90 fs after photoexcitation, the band crossing *E*_F_ is also extremely well described by an LL model, yielding a slightly reduced exponent of α = 0.67 ± 0.01 and an increased effective temperature *T*_e_ = 275 ± 11 K (Fig. 3B), associated with photoexcited electrons that have relaxed back to the Luttinger band. This indicates the emergence of a hot LL state, where the electron system has not yet thermalized with the lattice. We note that fitting transient spectra to equilibrium distribution functions has been widely adopted in pump-probe studies of quasi-2D and -3D materials with charge or lattice order (*27*–*30*), enabling extraction of the instantaneous chemical potential and electronic temperature. In our case, spectra show excellent agreement with an LL model at all delay times (Fig. 3C). A systematic assessment of the fitting reliability is provided in section S2. In Fig. 3D, we also plot the difference between the integrated electron distribution curves (EDCs) before and after laser excitation, which emphasizes the changes more clearly.

**Fig. 4.**
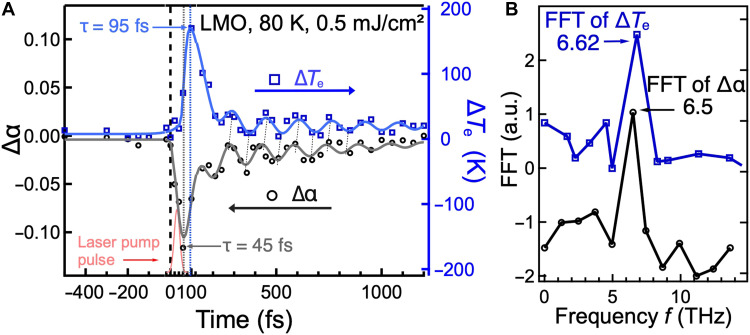
Ultrafast excitation and relaxation of the LL in LMO. (**A**) Evolution of the electron temperature and the Luttinger exponent α after laser excitation, for an initial sample temperature of 80 K. A clear lag is observed between when Δα (black circles) exhibits its maximum drop at ∼45 fs and when Δ*T*_e_ (blue squares) peaks at ∼90 fs. The solid lines are the fits using Eq. 2. (**B**) Fast Fourier transform (FFT) analysis of the data in (A), showing oscillation frequencies of 6.5 THz for Δα and 6.62 THz for Δ*T*_e_.

From these fits, we can plot the time evolution of α and *T*_e_ relative to their equilibrium values (Fig. 4A). Immediately following laser excitation, α drops by 14% within the 45-fs laser pump pulse, as electrons are transferred from the Luttinger band to unoccupied states above *E*_F_. Then, α rapidly recovers on a comparable timescale, likely reflecting electron relaxation back into a hot Luttinger band due to strong inelastic electron scattering, similar to that reported in conventional metals (*31*). Throughout the entire time window, α remains at all times well above 0.5, indicating that strong electron-electron interactions are preserved in the Luttinger band.

In contrast, the electron temperature *T*_e_ in the LL band rises with a noticeable time lag of about 45 to 50 fs, increasing from a base temperature of 80 K to a peak of ∼200 K at 90 fs and subsequently recovering within ∼200 fs. The increase in *T*_e_ coincides with the recovery of α, which indicates that higher-lying states in the Luttinger band are being filled because of the relaxation of excited electrons. After these very fast excitation and relaxation processes, coherent oscillations appear in both α and *T*_e_, starting within 100 fs (where *T*_e_ peaks) and persisting for nearly 1 ps. As we discuss below, the very fast cooling of *T*_e_, 10 times faster than in a normal Fermi metal, is due to the excitation of a collective nonequilibrium plasmon.

To quantitatively analyze these dynamics, we fit both Δα(*t*) and Δ*T*_e_(*t*) using a biexponential model combined with a damped cosine term

$$y=B_{1}e^{-∆t/\tau_{1}}+B_{2}e^{-∆t/\tau_{2}}\text{cos}\left(2\pi f∆t+\varphi \right)$$ (2)

where τ_1_ and τ_2_ are the rise and decay time constants, B_1,2_ is intensity, and the function is convolved with a Gaussian function of 45 fs full width at half maximum to account for the experimental temporal resolution. From the fits, we obtain timescales τ_1_ = 45 and 95 fs and τ_2_ = 98 and 115 fs for Δα and Δ*T*_e_, respectively. The fitted oscillation frequencies *f* are 6.50 ± 0.09 THz for Δα and 6.62 ± 0.12 THz for Δ*T*_e_ and are consistent with the Fourier transforms shown in Fig. 4B. These coherent oscillations indicate an ultrafast modulation of the band structure and the DOS, which we attribute to a collective plasmon excitation (see next section). This mechanism is reminiscent of band modulations driven by coherent amplitude modes in a quasi-2D charge density wave (CDW) material (*28*). The in-phase character of the oscillations in Δα and Δ*T*_e_ is qualitatively compatible with an isentropic modulation (see section S4): As the electronic system is effectively isolated from the lattice on sub-picosecond timescales, an increase of the Fermi velocity associated with a larger exponent α leads to a broadening of the electronic distribution and therefore to an increase of the electron temperature.

Last, we note that there is a ∼20-fs lag in the oscillations of *T*_e_ relative to α, suggesting that the response of the electronic distribution to the band modulation is delayed because of finite electron-electron scattering time and band filling. This timescale is in line with the short electron-electron scattering times expected in LMO, comparable to the intrachain tunneling time of about 5 fs (*13*). This collective behavior is distinct from conventional metals, highlighting the unique ultrafast many-body dynamics of an LL such as LMO.

## DISCUSSION

Our measurements uncover an ultrafast modulation of the electron-electron interactions in a quasi-1D LL. By tracking the time evolution of the Luttinger exponent α and electronic temperature *T*_e_ following femtosecond laser excitation, we reveal three key observations: (i) A rapid drop and subsequent recovery of α within ∼100 fs indicate depletion and refilling of the Luttinger band. (ii) This is accompanied by a delayed rise in *T*_e_ that reflects carrier relaxation back to the Luttinger band from higher-lying photoexcited states. In adition, (iii) synchronized oscillations in both α and *T*_e_, with a frequency of ∼6.6 THz with a relative time lag of ∼20 fs, indicative of a collective mode driving a periodic modulation of the electronic structure. The distinctively fast dynamics in LMO, enabled by strong electron-electron correlations and coherent plasmon excitations, contrast sharply with the far slower, electron-phonon or CDW-mediated interactions observed in materials such as Ta_2_NiSe_5_ (*32*) and TaSe_3_ (*33*).

To determine the origin of the coherent oscillations observed in both α and *T*_e_, we first assess alternative excitations based on their frequencies and coupling conditions. A weak phonon mode near 220 cm^−1^ (∼6.6 THz) has been reported in Raman studies at cryogenic temperatures (∼20 K) (*15*), but its extremely small amplitude and inefficient coupling to light limit its ability to be coherently excited. Moreover, such low-temperature phonons are unlikely to persist or maintain coherence at the elevated temperature of 80 K used in our experiment. Likewise, phasons from pinned CDWs are ruled out by the absence of long-range CDW order above ∼25 K (*34*). In contrast, a quench of electron density in the Luttinger band is likely to produce an electronic collective excitation because of the dominant intrachain Coulomb interactions in LMO (relative to, for example, electron-phonon coupling). Further, unlike Bloch oscillations of single electrons in a periodic band, which are strongly suppressed in 1D correlated conductors due to lack of quasiparticles, the oscillations we observe must stem from a collective bosonic excitation. These considerations support our assignment of the coherent oscillations to a 1D, nonequilibrium plasmon mode.

To gain theoretical insight into this collective mode, we model the LL as a 1D hydrodynamic system supplemented with the LL equation of state and coupled to an additional scalar variable Φ, which stands in for any additional df and interactions (see section S5). This approach is motivated by the relatively high excitation energies in our experiment, on the order of the Fermi energy *E*_F_, and applies after initial electron-electron scattering causes all nonconserved df to decay. The solutions of this model are collective modes with dispersion $\omega \left(k\right)=u_{0}k-i\;\gamma \;k^{2}$, which successfully reproduce the observed damping of oscillations at long times. A full quantitative description of the experimental data requires a detailed microscopic treatment that goes beyond the scope of this work. In particular, strong Coulomb interactions between the 1D chains—estimated to be *V* ∼ 0.5 eV (*35*) and comparable to intrachain interactions—are likely to affect the nature, dispersion, and decay of the nonequilibrium plasmon.

Qualitatively, the in-phase oscillations of *T*_e_ and α can be understood within two complementary frameworks. From the perspective of the LL DOS (Eq. 1), a modulation of the interaction parameter α redistributes spectral weight toward *E*_F_, resulting in a broadened electronic distribution and increased *T*_e_. Alternatively, from a thermodynamical perspective, this modulation of the LL state can be assumed isentropic because electrons are effectively isolated from the lattice on sub-picosecond timescales and their scattering rate is high relative to the frequency of the coherent oscillation. In 1D systems with linear dispersion, the entropy scales as $S\propto \frac{k_{s}T}{\mathrm{\hbar v}}L$, where v is the velocity of collective excitations and *L* is the system’s length. Because v increases with stronger repulsive interactions (larger α), an isentropic process would naturally cause *T*_e_ to rise with α (see section S4). Both pictures are consistent with the observed synchronized oscillations visible in Fig. 4A. The 20-fs phase delay between the oscillations of *T*_e_ and α may originate from the finite electron-electron scattering time that governs how quickly spectral weight redistributes around the Fermi level following a modulation of α.

Last, we note that it is well established that two coupled quasi-1D bands are present in LMO. It is possible that interactions between these Luttinger bands, or interchain interactions, may also contribute to the oscillations and damping observed experimentally. Future studies of interband scattering and interactions motivate more advanced many-body theories to develop a better understanding of such many body physics.

In summary, time- and ARPES measurements on LMO reveal that ultrafast laser excitation can coherently modulate electron-electron interactions in a prototypical LL. The dynamics are characterized by a very rapid drop and recovery of the Luttinger exponent α, within ∼100 fs. This is accompanied by a delayed rise and fall in the electronic temperature *T*_e_, as hot carriers scatter back to the Luttinger band from higher-lying photoexcited states. Subsequently, we observe long-lived (∼1 ps) coherent oscillations in both α and *T*_e_ at ∼6.6 THz, which we attribute to a nonequilibrium plasmon mode. A hydrodynamic model of a nonequilibrium LL coupled to an additional scalar df reproduces the essential features of these oscillations, including their damping and finite frequency. These results demonstrate that strong correlation effects in a 1D quantum metal can survive nonequilibrium conditions and that collective charge excitations can be launched and controlled on femtosecond timescales. More broadly, our findings highlight LMO as a promising platform for ultrafast manipulation of interaction-driven phenomena in low-dimensional quantum systems.

## MATERIALS AND METHODS

The time-resolved ARPES spectra are collected in a table-top beamline (*28*)*.* We use extreme-ultraviolet (EUV) light with photo energy of 22.4 eV and SPEC PHOIBOS 100 analyzer to record our photoelectrons. A 1.6-eV infrared laser pulse with a duration of 45 fs is generated from a Ti:Sapphire oscillator-amplifier system (KMLabs RAEA). It is then split into a pump beam to excite the sample and a probe line. The beam of the probe line is frequency doubled by a BBO to 3.2 eV and then focused into a waveguide getting high harmonic generation to provide the EUV. The overall energy resolution is about 80 meV, which is mainly limited by the bandwidth of the ultrashort laser pulses. Single crystals of LMO were cleaved in situ and measured under a vacuum of 1 × 10^−10^ torr at 80 K. The sample growth and basic characterization of LMO are given in Ke *et al.* (*36*). The measurements were performed using a low pump fluence chosen to minimize photoinduced broadening and to maintain the integrity of the LL spectral features. The fluence was verified to avoid space-charge artifacts and any detectable pump-induced energy shifts.

## Supplementary Materials

### This PDF file includes:

Sections S1 to S9 Figs. S1 to S6 References
