# Supplementary material for: Femtosecond modulation of electron correlations in a Luttinger liquid
Source: Sci Adv. 2026 May 27;12(22):eaec7494. doi: 10.1126/sciadv.aec7494 (PMC13215176; doi:10.1126/sciadv.aec7494)
Supplement: Supplementary file 1 — Sections S1 to S9 Figs. S1 to S6 References [file sciadv.aec7494_sm.pdf]

Science Advances
AAAS

Supplementary Materials for

Femtosecond modulation of electron correlations in a Luttinger liquid

Na Li *et al.*

Corresponding author: Na Li, na.li-1@colorado.edu

Sci. Adv. **12**, eaec7494 (2026)
DOI: 10.1126/sciadv.aec7494

This PDF file includes:

Sections S1 to S9
Figs. S1 to S6
References

Section S1. LL behavior in Quasi-1D $L_{0.9}Mo_6O_{17}$

Figure S1, A and B show the electronic band structures of $Li_{0.9}Mo_6O_{17}$ (LMO) along the Γ–Y and an off-normal cut, measured at 80 K before time-zero in TR-ARPES. The spectra are consistent with previous static ARPES measurements *(3, 11)* and calculated band structures *(21)*. To quantify the dispersions, a series of momentum distribution curves (MDCs) were taken at energies from -0.18 eV to 0.12 eV [fig. S1, C and D]. The MDC peak positions (black dotted lines) indicate a nearly linear band crossing the Fermi level. Two Mo-4d bands become degenerate near -0.13 eV binding energy and then disperse linearly through E_F together, consistent with Luttinger-liquid (LL) behavior *(11)*.

Temperature-dependent integrated energy distribution curves (EDCs) at $k \approx k_F$ (~0.29 $Å^{-1}$) along Γ–Y [fig. S1E] are well captured by finite-temperature LL spectral functions (red lines). The extracted LL exponent α [fig. S1F] shows a pronounced temperature dependence, with a typical fitting error bar of ±0.01. It decreases from ~ 0.85 at 300 K to ~ 0.5 at 150 K, then rises to ~ 0.73 at 80 K — consistent with previous reports (1) and indicative of strong correlations and non-Fermi-liquid behavior in quasi-1D LMO.

Section S2. LL model fitting of integrated EDCs

We analyze the EDCs using the finite-temperature Luttinger liquid (LL) model [Eq. (1) in the main text].

$$\rho(\varepsilon, \alpha, T_e) \propto T_e^{\alpha} Re\left[(2i)^{\alpha+1} B\left(\frac{\alpha+1-i\varepsilon/\pi}{2}, -\alpha\right)\right] \quad \text{(S1)}$$

We also have a scaling factor and background, which are free fit parameters. The energy window used for fitting is from –0.18 eV to 0.12 eV relative to E_F at a momentum point close to k_F, which is a prerequisite for applying the LL model. The measured temperature is corrected as *(28)* $T_e = \sqrt{T^2 - T_{res}^2}$, where T_{res} is determined by requiring T = 300 K when measured at room temperature before laser excitation.

1. The reliability of the extracted LL exponent α and temperature T is crucial for interpreting the dynamics: uncertainty of α. Using the –500 fs data as a benchmark, we find that α can be determined with high precision. As shown in fig. S2A, even small deviations (~0.02) from the optimal α lead to a clear deviation from experimental data. This sensitivity confirms the robustness of the extracted α values and the LL behavior in LMO.

2. Uniqueness of fitting parameters**.** At each time delay, only a narrow range of (T, α) yields a good fit. For example, at –500 fs and base temperature 80 K, we obtain α = 0.73 and T = 90 K. Small changes in T within ± 11 K (~10%) can be compensated by a slight adjustment in α, but larger deviations cannot be matched by any α. This demonstrates the inherent stability of our fitting procedure.

Section S3. Evolution of the α and T_e

We fit the dynamics of α and T_e with an equation combining two-exponential functions which account for the excitation and relaxation, and a damped-oscillation function:

$$y = B_1 * e^{-\Delta t/\tau_1} + B_2 * e^{-\Delta t/\tau_2} * \cos(2\pi f * \Delta t + \varphi) \quad \text{(S2)}$$

where τ_1 and τ_2 are the exponential rise and decay constants. And $B_{1,2}$ is intensity. The temporal resolution is considered by convoluting the fitting function with a Gaussian function (with full width at half maximum of 45 fs).

Section S4. In-phase modulation of α and T_e in an adiabatic transformation

In this Section, we detail the relation between temperature and Luttinger exponent under the assumption that the electron gas undergoes a quasi-static, adiabatic transformation due to the oscillation of a collective mode on ~200 –1000 fs timescales. The entropy of a one-dimensional system with linear dispersion can generally be written (up to a proportional constant) as

$$S(T) \propto \frac{k_B T}{\hbar v} L \tag{S3}$$

Where T is the temperature, v is the velocity of collective excitations, and L is the system's length. As a result, the ratio T/v is constant for an isentropic transformation.

In a Luttinger liquid, the charge excitation velocity is related to the Fermi velocity v_F as $v = v_F/K_\rho$, where K_ρ is the Luttinger parameter associated with the charge degree-of-freedom *(8)*. The Luttinger parameter is in turn related to the Green's function exponent α in a spin-rotation invariant system such as LMO through *(13, 27)*:

$$\alpha = \frac{1}{4}\left(K_\rho + K_\rho^{-1}\right) - \frac{1}{2} \tag{S4}$$

Solving this equation results in two possible values $K_\rho^{\pm} = 2\alpha \pm \sqrt{(2\alpha+1)^2 - 1}$. For repulsive Coulomb interactions, $K_\rho = K_\rho^- < 1$, and is a monotonically decreasing function of α.

Within this framework, a pump-induced reduction of the effective electron density on <50 fs timescales enhance repulsive interaction effects, which leads to a decrease of the Luttinger parameter K_ρ and the anomalous exponent α, and in turn increases the renormalized charge excitation velocity v. Then, as the system cools on ~ 200 – 1000 fs timescales, under the assumption of an adiabatic, isentropic evolution of the electron gas, this increases in v results in a simultaneous increase in the effective electronic temperature. This trend is qualitatively compatible with the in-phase oscillations of α and T_e observed in Fig. 3.

Section S5. Hydrodynamic model for the collective mode

The energy scales on which the electron occupancy in the sample is excited are large: of the order of the Fermi energy E_F. A theoretical model of the physics of the observed collective mode hence requires non-equilibrium techniques. We demonstrate, in this section, that a first-order hydrodynamic model of the Luttinger liquid with minimal assumptions produces such a decaying collective mode. The degrees of freedom are the electron density n of the Luttinger band, the electron velocity v (or equivalently the electron momentum $\pi=nv$), and the energy density ε. Furthermore, we introduce a scalar field Φ as a qualitative stand-in for any additional degrees of freedom that interact with the Luttinger band. These could be coming from other bands or from phonons, the details of which we ignore since they must be small on few hundred femtosecond timescales. More precise modelling of these additional degrees of freedom may change the small details of the observed oscillation, but not the qualitative picture behind the physics that governs its existence. The hydrodynamic equations take the following form

$$\begin{gathered}\partial_t n + \partial_x j = 0 \\ \partial_t (nv) + \partial_x T^{xx} = 0 \\ \partial_t \left(\varepsilon + p + \frac{nv^2}{2}\right) + \partial_x j_\varepsilon = 0 \\ \partial_t^2 \Phi + v_\Phi^2 \partial_x^2 \Phi = \gamma_n \partial_x^2 \mu + \gamma_\varepsilon \partial_x^2 T + \gamma_\pi \partial_x (v \partial_x v)\end{gathered} \tag{S5}$$

Where μ and T are the electron chemical potential and temperature, p is the electron pressure, v_Φ is the velocity of the degree of freedom Φ, γ_n, γ_ε, d, γ_π are response coefficients between the scalar field and the hydrodynamic variables, and j, T^{xx}, j_ε are the electron current, stress tensor and energy current defined by the first-order constitutive relations

$$\begin{aligned} j &= nv - \gamma_n \partial_x \mu_\Phi \\ T^{xx} &= p + nv^2 - \xi \partial_x v - \gamma_\pi v \partial_x \mu_\Phi \\ j_\varepsilon &= \left(\varepsilon + p + \frac{nv^2}{2}\right) v - \xi v \partial_x v - \kappa \partial_x T - \gamma_\varepsilon \partial_x \mu_\Phi \end{aligned} \tag{S6}$$

In the above we have used Onsager reciprocity to write down the terms proportional to $\partial_x \mu_\Phi$. The Greek symbols above denote transport coefficients, with ξ being the viscosity of the electron fluid and κ, the thermal conductivity. These equations are to be supplemented by various thermodynamic relations that determine the chemical potentials μ and μ_Φ, the energy density ε, and the electron pressure p, in terms of the electron density n, temperature T, and velocity v. These relations can be derived from the Luttinger liquid Hamiltonian as well as the Hamiltonian for Φ, given below

$$\beta H_{LL} = \frac{u}{2} \int_{t,x} \frac{1}{K} (\Pi + \mu)^2 + K(\partial_x \varphi)^2 \tag{S7}$$

$$\beta H_\Phi = \frac{1}{2} \int_{t,x} (\Pi_\Phi + \mu_\Phi)^2 + v_\Phi^2 (\partial_x \Phi)^2 \tag{S8}$$

In the above we have defined u as the Luttinger liquid velocity (not to be confused with the hydrodynamic velocity v), and K as the Luttinger parameter that encodes forward and backscattering. φ is the Luttinger liquid's low energy degree of freedom. The thermal free energies for the Luttinger liquid and the scalar field are then given by Matsubara sums

$$F_{LL} = T \sum_{\Omega_n, q} \log \frac{v}{2} \left(\frac{(\Omega_n + \mu)^2}{K} + K q^2 \right) \tag{S9}$$

$$F_\Phi = T \sum_{\Omega_n, q} \log \frac{(\Omega_n + \mu)^2 + q^2}{2} \tag{S10}$$

where $\Omega_n = 2\pi n T$ are Matsubara frequencies. The thermodynamic relations can be straightforwardly computed from the free energies by taking derivatives with respect to the chemical potential, temperature and volume. Plugging these into the hydrodynamic equations gives us four equations for four variables, which we then linearize around a constant background by expanding $n = n_0 + \delta n,\ T = T_0 + \delta T$, and by taking v and Φ to be small. After linearizing and Fourier transforming to frequency $\omega \sim i\partial_t$ and momentum $q \sim -i\partial_x$, the equations take the following matrix form

$$\begin{pmatrix} \omega & -n_0 q & 0 & A\omega q^2 \\ Bq & -(n_0\omega + i\xi q^2) & 2k_F q & 0 \\ D\omega - Bq & 0 & E\omega - 2k_F q + i\kappa q^2 & F\omega q^2 \\ Gq^2 & 0 & Hq^2 & v_\Phi^2 q^2 - \omega^2 \end{pmatrix} \begin{pmatrix} \delta n \\ v \\ \delta T \\ \Phi \end{pmatrix} = 0 \tag{S11}$$

Above we have defined various constants A through H in terms of hydrodynamic coefficients as well as Luttinger liquid parameters

$$A = \frac{\gamma_n}{C'T_0}, \qquad B = Kcn_0, \qquad D = \frac{\pi c' E_F v_F T_0}{K}$$

$$E = \frac{c' E_F^2}{2K}, \quad F = \frac{\gamma_\varepsilon}{C'T_0}, \quad G = \frac{\gamma_n}{CT_0}, \quad H = \gamma_\varepsilon - \frac{\gamma_n n_0}{CT_0^2}$$

and *c, c', C, C'* are numerical constants. E_F, k_F, v_F are the Fermi energy, momentum and velocity, respectively. A solution of the equations above exists only if the determinant of the 4x4 matrix vanishes. The vanishing condition provides the dispersion relation of the collective modes that this model admits. The determinant can further be expanded in a power series in q, and we find that it admits a solution with the following dispersion relation

$$\omega = uq - i\Gamma q^2 \tag{S12}$$

where the speed u and the damping Γ are given by the following equations

$$u = \frac{k_F}{E} + \sqrt{\frac{k_F^2}{E^2} - \frac{2Dk_F}{E} + B} \tag{S13}$$

$$\Gamma = \frac{(u^2 - v_\Phi^2)}{n_0} \frac{[u(\kappa n_0 u - 2k_F \xi + E\xi u) - B\kappa n_0]}{(3u^2 - v_\Phi^2)(2Dk_F - BE) + u(5Eu^3 - 8k_F u^2 - 3Euv_\Phi^2 + 4k_F v_\Phi^2)} \tag{S14}$$

The Fourier transform of such a solution has the shape of the decaying oscillation observed in the experiment.

We emphasize again that this calculation presents the qualitative physics underlying the observed decaying oscillation, i.e., linearized hydrodynamics, but doesn't make predictions about the quantitative details of the shape of its dispersion, owing to the various undetermined couplings and transport coefficients used in the hydrodynamic model. A full-fledged quantitative description would require a microscopic calculation of these effective couplings in terms of the microscopic ones, and a more detailed modelling of the degrees of freedom coming from outside the Luttinger band.

Section S6. Calculated band structure of $Li_{0.9}Mo_6O_{17}$

First-principles calculations of the electronic structure and its time evolution was performed using the Elk software *(37)*. The exchange and correlation was treated within the local density approximation as parametrized by Perdew and Wang *(38, 39)*. Time-dependent density-functional theory was used to estimate the direct influence of light on the band-structure. Bear in mind that the dynamics due to the strong correlation of Luttinger liquids are not well represented by semi-local density functional theory, hence the relaxation process is not directly available for analysis.

Figure S3 shows the calculated band-structure, in panel (A) we estimate the renormalization by investigating the energies of the Houston states $\epsilon_i(t)$, as defined through the instantaneous eigenstate of the time-dependent Hamiltonian *(40, 41)*:

$$H(t) = \left[\tfrac{1}{2} \left(-i\nabla + \frac{A_{\mathrm{ext}}(t) + A_{ind}(t)}{c} \right)^2 + v_s(r,t) + \frac{\sigma \cdot B_s(r,t)}{2c} + \frac{\sigma \cdot (\nabla v_s(r,t) \times (-i\nabla))}{4c^2} \right] \tag{S15}$$

Where $A_{\mathrm{ext}}(t)$ is the applied vector potential, $A_{ind}(t)$ is the induced vector potential, $v_s(r,t)$ is the scalar Kohn-Sham potential, $B_s(r,t) = B_{ext}(t) + B_{xc}(r,t)$ is the effective Kohn-Sham magnetic field, the final term represents the spin-orbit coupling within the time-dependent Kohn-Sham Hamiltonian. The width of the red region is calculated from the expectation value of the standard deviation of the instantaneous eigenvalue, $\sigma = \left\langle \sqrt{\langle \epsilon_i^2(t) \rangle - (\langle \epsilon_i(t) \rangle)^2} \right\rangle_i$, the subscript i for the outer expectation value indicates an average over all valence bands, calculated for a Γ-centered k-point grid of dimension 4x3x4. The renormalization is in the order of 0.05 eV, which is in the same range as the

thermal broadening. The. Panel (B) shows the spectral representation of the band-structure in the Brillouin-zone cut of $Y - \Gamma - Y$, with a thermal broadening corresponding to 80 K.

Section S7. Evolution of the photoemission spectra and electron intensity

Our detailed ARPES measurements demonstrate that the dynamics and oscillatory behavior are directly observable in the raw data, not just in our LL fits. The time evolution of the photoemission spectra (fig. S4A) clearly reveals fast dynamics associated with the LL band response and oscillations characteristic of plasmon excitation. The transient ARPES intensity above E_F (fig. S4B) shows a distinct rise followed by a fast recovery, with oscillations that are evident in the subtracted integrated intensity (fig. S4C). A comparison of the transient ARPES intensity integrated over [0.05, 0.25] eV above E_F and [-0.15, -0.05] eV below E_F reveals identical dynamics (fig. S4D). Following laser excitation, the intensity shows an extended initial rise in the [0.05, 0.25] eV range (or corresponding drop in the [-0.15, -0.05] eV range) lasting approximately 80 fs (τ = 80 fs, marked by black dotted vertical line in fig. S4D). This is followed by rapid exponential recovery, reflecting the creation and relaxation of hot electrons and holes. Besides, the oscillatory behavior emerges at approximately 120 fs and persists up to 1 ps with damping. Fitting analysis and FFT confirm oscillations at ~6 THz. We observe that the oscillation frequencies in the intensities differ slightly from those of α and T_e. This discrepancy arises from various contributing processes, particularly near E_F, including electron thermalization effects at the Fermi surface, electron scattering, and the relaxation of high-energy electrons to lower energy states.

Section S8. Assessment of surface photovoltage and pump-induced energy shifts

To assess the possible influence of surface photovoltage (SPV) or pump-induced charging effects, we present in fig. S5 the time–dependent ARPES spectra over an extended binding-energy range, including the bottom of the valence band. The data are shown for a representative momentum cut near k_F and span large regions.

Panels (A) and (B) compare the photoemission spectra measured before pump excitation and at a representative pump–probe delay of 90 fs at 80 K. The position of the deep valence-band states remains stationary within the experimental energy resolution. No rigid shift of the band structure is observed following photoexcitation. This behavior rules out the presence of pump-induced SPV or global electrostatic potential changes, which would necessarily manifest as a uniform energy shift of all spectral features, including deep valence states and the Fermi edge.

Time-dependent photoemission spectra extracted from the momentum region indicated in fig. S5A are shown for selected energy regions above E_F, below E_F, and at the bottom of the valence band (fig. S5C–E). We can observe the obvious dynamics in energy regions above E_F, below E_F but barely in the bottom of the valence band. The corresponding transient ARPES intensities integrated over representative energy windows are summarized in fig. S5F, which show us a little change without oscillation in energy region near the bottom of the valence band.

In addition, pump-fluence–dependent tests were performed prior to acquiring the full dataset by monitoring the spectra at negative delays while gradually increasing the pump fluence. Such measurements are highly sensitive to SPV and space-charge effects. No systematic energy shift was detected, and the pump fluence used in the main experiments was chosen well below the threshold at which charging effects become apparent.

Section S9. Scaling analysis of integrated EDCs before and after photoexcitation

Regarding the scaling behavior, we replotted the integrated EDCs before pump excitation and at a pump–probe delay of 90 fs, shown in Fig. 3A of the main text, as a function of the dimensionless energy $(E - E_F)/k_B T_e$, where T_e is the effective electronic temperature independently extracted for each time delay. This representation allows a direct comparison of the spectral line shapes on a common reduced energy scale.

As shown in fig. S6, the equilibrium (80 K) and pump-induced spectra do not collapse onto a single curve in this representation. This indicates that the two spectra cannot be described by a single, common anomalous exponent α. Instead, the observed behavior is consistent with a pump-induced modification of the interaction parameters governing the Luttinger-liquid response, rather than a universal scaling behavior with a fixed α.

We note that the laser pump-induced reduction of α is qualitatively similar to the trend observed upon increasing temperature in equilibrium measurements, reflecting hot-electron broadening of the Luttinger-liquid spectral function. However, the transient response does not correspond to a one-to-one mapping onto an equilibrium temperature increase, because of the novel dynamics also observed.

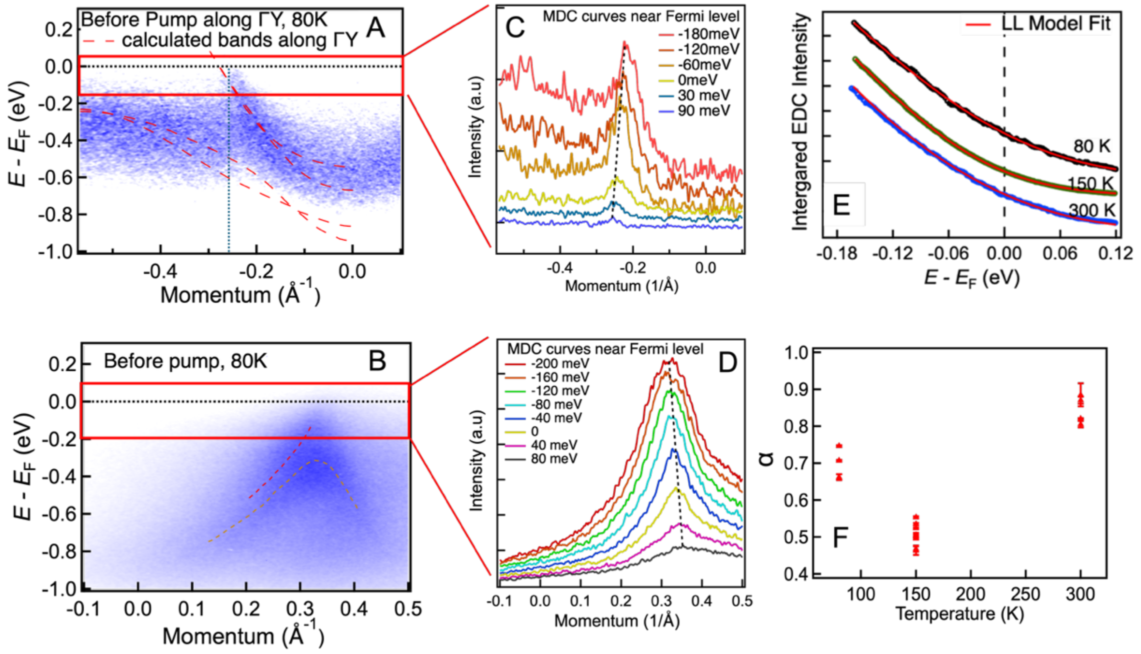

Fig. S1. Static ARPES band structure and its LL behavior. (**A**) Band structure taken at 80 K along $\Gamma - Y$ overlaid with calculated bands (red dashed lines) before pump. (**B**) Band structure taken at 80 K along off-normal direction. (**C**) and (**D**) show a nearly linear band on A and B from momentum distribution curves (MDCs) in the vicinity of the Fermi level (-0.18 ~ 0.12 eV). (**E**) shows *T*-dependent spectra angle integrated around k_F in (A) with finite LL fits. (**F**) shows an extracted Luttinger exponent α from fitting of (E) measured in three different samples at temperatures T = 80, 150 and 300 K. Typical errors from fitting are $\pm$ 0.01. The lowest α ~ 0.5 is obtained at 150 K, while stronger correlations α = 0.73 to 0.8 are found at 80 K or 300 K. Points in (E) indicate results from different samples. The vertical bule dotted line in A shows the momentum position for time-dependent photoemission spectrum in fig. S4.

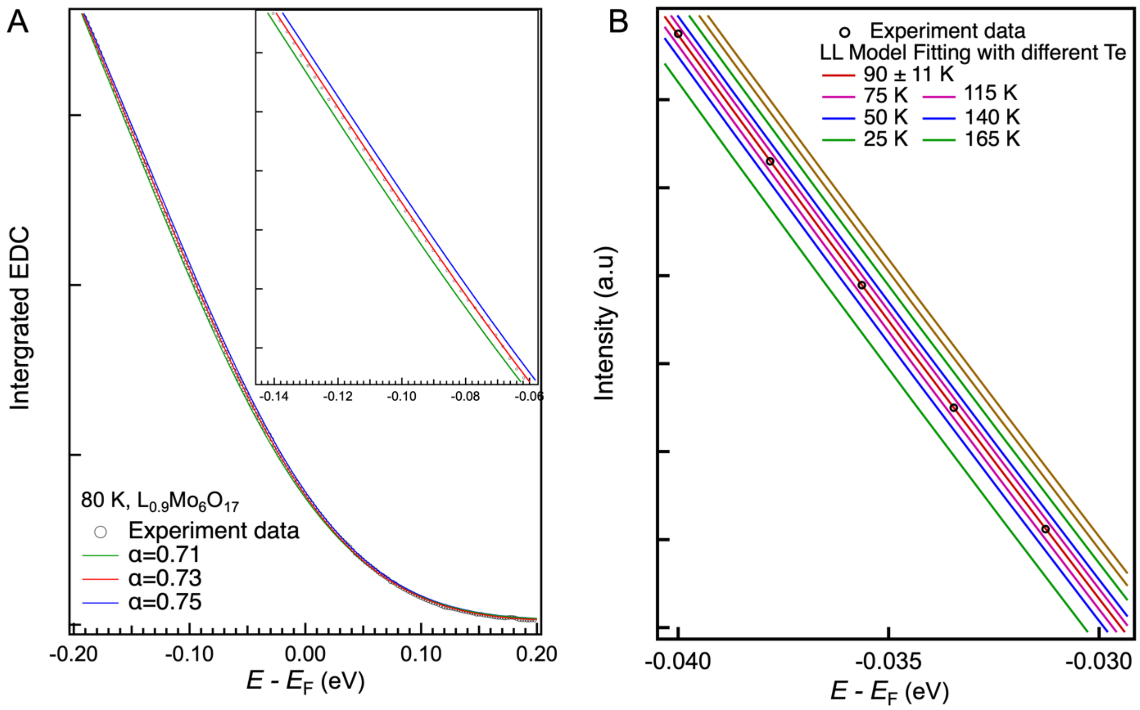

Fig. S2. Quantifying the uncertainty of the LL fits, confirming the robustness of the extracted LL parameters. (**A**). Fit sensitivity to the LL exponent α: red line is the optimal fit at –500 fs, others show α ± 0.02 deviations, which degrade the fit quality. (**B**). Uncertainty of the extracted electronic temperature T_e: red lines indicate the error margin, limited to 11 K.

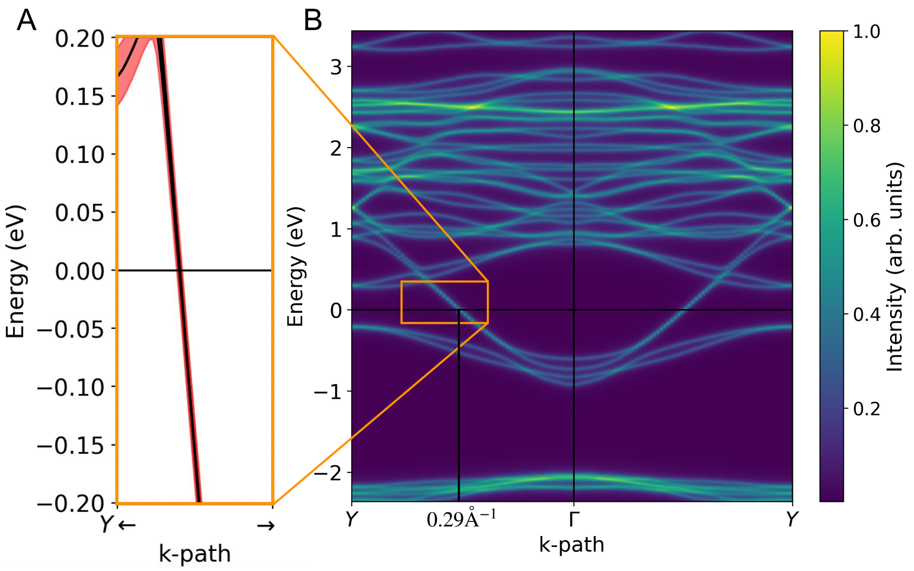

Fig. S3. The calculated band structure of $Li_{0.9}Mo_6O_{17}$ within the local density approximation as parametrized by Perdew and Wang based on quantum monte-carlo data of Ceperley and Alder. Panel (**A**) shows the details of the band-structure in the region of the Fermi energy and wave-vector. The red region corresponds to the average renormalization of the band-energies estimated from the energy of the Houston states. Panel (**B**) shows the band-structure in a spectral function representation, where the broadening corresponds to an electronic temperature of 80 K. The renormalization is on a similar energy-scale as the thermal broadening. The calculated bands agree well with literature *(42)*.

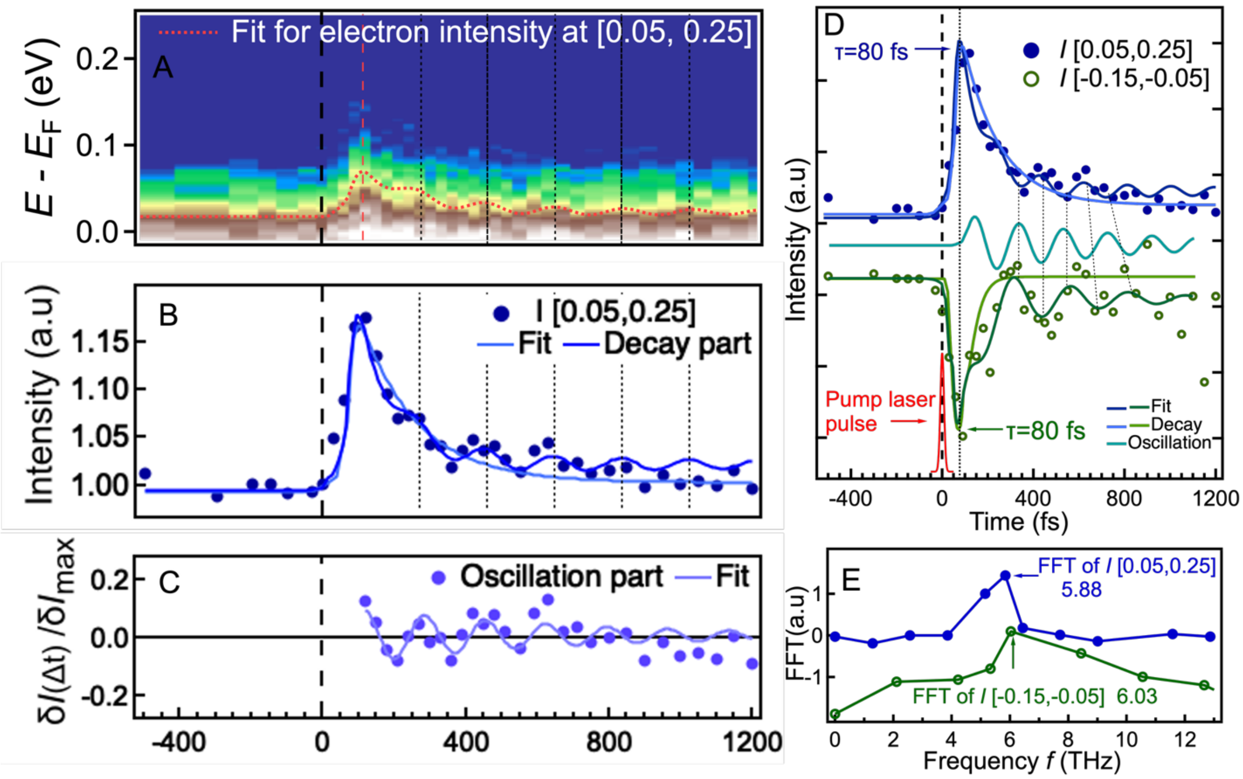

Fig. S4. Evolution of the band and the ARPES spectra. (A) Time-dependent photoemission spectrum at momentum $k_{//}$ as indicated by the vertical blue dotted lines in fig. S1A. The red and black dots are guidelines. (B) Time-dependent ARPES intensity (dark blue dots) in an energy window of [0.05,0.25] eV above E_F. The blue and light blue solid lines are the full fit using equation (S1) and fit including only the rise and fast-decay components, respectively. (C) Subtracted integrated intensity, identifying oscillation part with fit. (D) Comparison of integrated intensity on the energy window of [0.05, 0.25] eV and [-0.15, -0.05] eV, respectively. The lines are fits. (E) Fourier transform (FT) of the data in (D). Both fits and the FFT connect the oscillations to the ~6 THz plasmon**.**

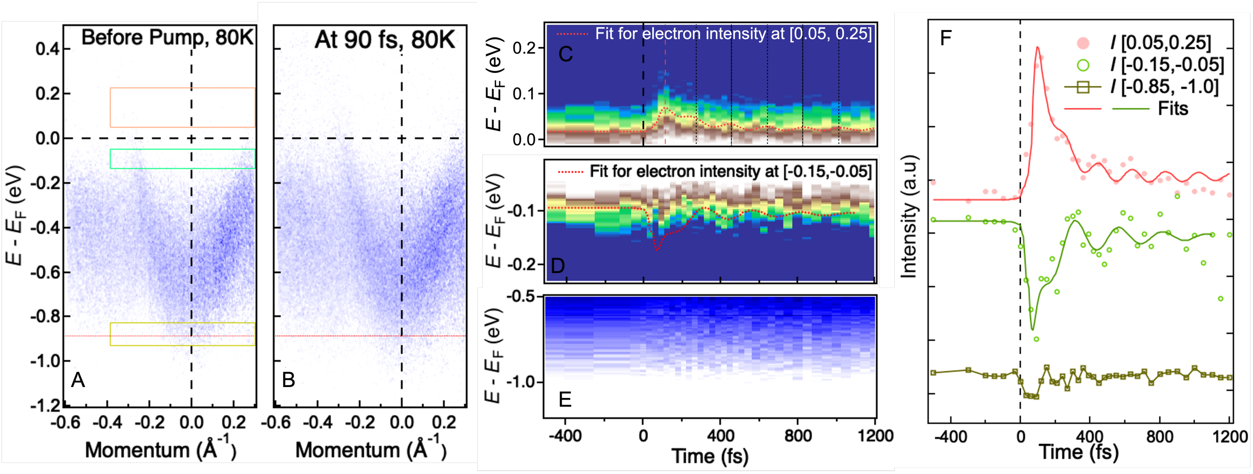

Fig. S5. Wide-energy-range time-dependent ARPES spectra and transient intensities in different energy regions. (A, B) Comparison of photoemission spectra measured before pump excitation and at a delay time of 90 fs at 80 K. (C–E) Time-dependent photoemission spectra extracted from the momentum region indicated by the box in (A), shown for selected energy regions above E_F (C), below E_F (D), and at the bottom of the valence band (E). (F) Transient ARPES intensities integrated over representative energy windows. The stationary position of deep valence states throughout the delay scan confirms the absence of pump-induced rigid energy shifts.

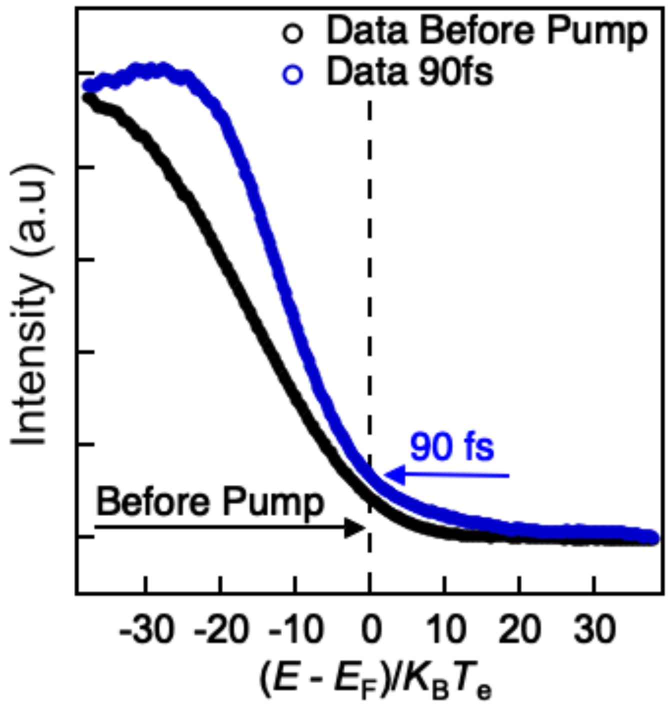

Fig. S6. **Scaling analysis of integrated EDCs before and after photoexcitation.** The same integrated ARPES EDCs shown in Fig. 3A, replotted as a function of the dimensionless energy $(E - E_F)/k_B T_e$.

REFERENCES

1. J. Liu, Y. Ding, M. Zeng, L. Fu, Chemical insights into two-dimensional quantum materials. *Matter* **5**, 2168–2189 (2022).

2. Z. Zhang, X. Liu, J. Yu, Y. Hang, Y. Li, Y. Guo, Y. Xu, X. Sun, J. Zhou, W. Guo, Tunable electronic and magnetic properties of two-dimensional materials and their one-dimensional derivatives. *WIREs Comput. Mol. Sci.* **6**, 324–350 (2016).

3. J. W. Allen, Quasi-particles and their absence in photoemission spectroscopy. *Solid State Commun.* **123**, 469–487 (2002).

4. S. Mathias, S. Eich, J. Urbancic, S. Michael, A. V. Carr, S. Emmerich, A. Stange, T. Popmintchev, T. Rohwer, M. Wiesenmayer, A. Ruffing, S. Jakobs, S. Hellmann, P. Matyba, C. Chen, L. Kipp, M. Bauer, H. C. Kapteyn, H. C. Schneider, K. Rossnagel, M. M. Murnane, M. Aeschlimann, Self-amplified photo-induced gap quenching in a correlated electron material. *Nat. Commun.* **7**, 12902 (2016).

5. J. Voit, "A brief introduction to Luttinger liquids," in *AIP Conference Proceedings* (American Institute of Physics, 2000), vol. 544, pp. 309–318.

6. G.-H. Gweon, J. W. Allen, J. D. Denlinger, Generalized spectral signatures of electron fractionalization in quasi-one- and two-dimensional molybdenum bronzes and superconducting cuprates. *Phys. Rev. B* **68**, 195117 (2003).

7. X. Du, L. Kang, Y. Y. Lv, J. S. Zhou, X. Gu, R. Z. Xu, Q. Q. Zhang, Z. X. Yin, W. X. Zhao, Y. D. Li, S. M. He, D. Pei, Y. B. Chen, M. X. Wang, Z. K. Liu, Y. L. Chen, L. X. Yang, Crossed Luttinger liquid hidden in a quasi-two-dimensional material. *Nat. Phys.* **19**, 40–45 (2023).

8. T. Giamarchi, *Quantum Physics in One Dimension* (Oxford Univ. Press, 2003).

9. R. Claessen, M. Sing, U. Schwingenschlögl, P. Blaha, M. Dressel, C. S. Jacobsen, Spectroscopic signatures of spin-charge separation in the quasi-one-dimensional organic conductor TTF-TCNQ. *Phys. Rev. Lett.* **88**, 096402 (2002).

10. M. Bockrath, D. H. Cobden, J. Lu, A. G. Rinzler, R. E. Smalley, L. Balents, P. L. McEuen, Luttinger-liquid behaviour in carbon nanotubes. *Nature* **397**, 598–601 (1999).

11. F. Wang, J. V. Alvarez, S.-K. Mo, J. W. Allen, G.-H. Gweon, J. He, R. Jin, D. Mandrus, H. Höchst, New Luttinger-liquid physics from photoemission on $Li_{0.9}Mo_6O_{17}$. *Phys. Rev. Lett.* **96**, 196403 (2006).

12. L. Kang, X. Du, J. S. Zhou, X. Gu, Y. J. Chen, R. Z. Xu, Q. Q. Zhang, S. C. Sun, Z. X. Yin, Y. W. Li, D. Pei, J. Zhang, R. K. Gu, Z. G. Wang, Z. K. Liu, R. Xiong, J. Shi, Y. Zhang, Y. L. Chen, L. X. Yang, Band-selective Holstein polaron in Luttinger liquid material $A_{0.3}MoO_3$ (A = K, Rb). *Nat. Commun.* **12**, 6183 (2021).

13. P. Chudzinski, T. Jarlborg, T. Giamarchi, Luttinger-liquid theory of purple bronze $Li_{0.9}Mo_6O_{17}$ in the charge regime. *Phys. Rev. B* **86**, 075147 (2012).

14. J. L. Cohn, P. Boynton, J. S. Triviño, J. Trastoy, B. D. White, C. A. M. Dos Santos, J. J. Neumeier, Stoichiometry, structure, and transport in the quasi-one-dimensional metal $Li_{0.9}Mo_6O_{17}$. *Phys. Rev. B* **86**, 195143 (2012).

15. M. K. Tran, A. Akrap, J. Levallois, J. Teyssier, P. Schouwink, C. Besnard, P. Lerch, J. W. Allen, M. Greenblatt, D. van der Marel, Pressure-induced structural transitions triggering dimensional crossover in the lithium purple bronze $Li_{0.9}Mo_6O_{17}$. *Phys. Rev. B* **103**, 235124 (2021).

16. C. A. M. Dos Santos, B. D. White, Y.-K. Yu, J. J. Neumeier, J. A. Souza, Dimensional crossover in the purple bronze $Li_{0.9}Mo_6O_{17}$. *Phys. Rev. Lett.* **98**, 266405 (2007).

17. P. Wang, G. Yu, Y. H. Kwan, Y. Jia, S. Lei, S. Klemenz, F. A. Cevallos, R. Singha, T. Devakul, K. Watanabe, T. Taniguchi, S. L. Sondhi, R. J. Cava, L. M. Schoop, S. A. Parameswaran, S. Wu, One-dimensional Luttinger liquids in a two-dimensional moiré lattice. *Nature* **605**, 57–62 (2022).

18. C. Blumenstein, J. Schäfer, S. Mietke, S. Meyer, A. Dollinger, M. Lochner, X. Y. Cui, L. Patthey, R. Matzdorf, R. Claessen, Atomically controlled quantum chains hosting a Tomonaga–Luttinger liquid. *Nat. Phys.* **7**, 776–780 (2011).

19. A. Schwartz, M. Dressel, G. Grüner, V. Vescoli, L. Degiorgi, T. Giamarchi, On-chain electrodynamics of metallic (TMTSF)2X salts: Observation of Tomonaga-Luttinger liquid response. *Phys. Rev. B* **58**, 1261–1271 (1998).

20. Z. S. Popović, S. Satpathy, Density-functional study of the Luttinger liquid behavior of the lithium molybdenum purple bronze $Li_{0.9}Mo_6O_{17}$. *Phys. Rev. B* **74**, 045117 (2006).

21. G.-H. Gweon, S.-K. Mo, J. W. Allen, J. He, R. Jin, D. Mandrus, H. Höchst, Luttinger liquid angle-resolved photoemission line shapes from samples of $Li_{0.9}Mo_6O_{17}$ grown by the temperature-gradient-flux technique. *Phys. Rev. B* **70**, 153103 (2004).

22. G.-H. Gweon, J. D. Denlinger, C. G. Olson, H. Höchst, J. Marcus, C. Schlenker, Photoemission view of electron fractionalization in quasi-one-dimensional metal $Li_{0.9}Mo_6O_{17}$. *Phys. B Condens. Matter* **312-313**, 584–585 (2002).

23. L. Dudy, J. D. Denlinger, J. W. Allen, F. Wang, J. He, D. Hitchcock, A. Sekiyama, S. Suga, Photoemission spectroscopy and the unusually robust one-dimensional physics of lithium purple bronze. *J. Phys. Condens. Matter* **25**, 014007 (2013).

24. J. Hager, R. Matzdorf, J. He, R. Jin, D. Mandrus, M. A. Cazalilla, E. W. Plummer, Non-Fermi-liquid behavior in quasi-one-dimensional $Li_{0.9}Mo_6O_{17}$. *Phys. Rev. Lett.* **95**, 186402 (2005).

25. G.-H. Gweon, J. D. Denlinger, J. W. Allen, R. Claessen, C. G. Olson, H. Höchst, J. Marcus, C. Schlenker, L. F. Schneemeyer, ARPES line shapes in FL and non-FL quasi-low-dimensional inorganic metals. *J. Electron Spectrosc. Relat. Phenom.* **117-118**, 481–502 (2001).

26. D. Orgad, Spectral functions for the Tomonaga-Luttinger and Luther-Emery liquids. *Philos. Mag. B* **81**, 377–398 (2001).

27. Y. Zhang, X. Shi, W. You, Z. Tao, Y. Zhong, F. Cheenicode Kabeer, P. Maldonado, P. M. Oppeneer, M. Bauer, K. Rossnagel, H. Kapteyn, M. Murnane, Coherent modulation of the electron temperature and electron-phonon couplings in a 2D material. *Proc. Natl. Acad. Sci. U.S.A.* **117**, 8788–8793 (2020).

28. X. Shi, W. You, Y. Zhang, Z. Tao, P. M. Oppeneer, X. Wu, R. Thomale, K. Rossnagel, M. Bauer, H. Kapteyn, M. Murnane, Ultrafast electron calorimetry uncovers a new long-lived metastable state in 1T-$TaSe_2$ mediated by mode-selective electron-phonon coupling. *Sci. Adv.* **5**, eaav4449 (2019).

29. S. Duan, W. Xia, C. Huang, S. Wang, L. Gu, H. Liu, D. Xiang, D. Qian, Y. Guo, W. Zhang, Ultrafast switching from the charge density wave phase to a metastable metallic state in 1T-$TiSe_2$. *Phys. Rev. Lett.* **130**, 226501 (2023).

30. F. Y. Gao, Z. Zhang, Z. Sun, L. Ye, Y.-H. Cheng, Z.-J. Liu, J. G. Checkelsky, E. Baldini, K. A. Nelson, Snapshots of a light-induced metastable hidden phase driven by the collapse of charge order. *Sci. Adv.* **8**, eabp9076 (2022).

31. M. Bauer, A. Marienfeld, M. Aeschlimann, Hot electron lifetimes in metals probed by time-resolved two-photon photoemission. *Prog. Surf. Sci.* **90**, 319–376 (2015).

32. D. Golež, S. K. Y. Dufresne, M.-J. Kim, F. Boschini, H. Chu, Y. Murakami, G. Levy, A. K. Mills, S. Zhdanovich, M. Isobe, H. Takagi, S. Kaiser, P. Werner, D. J. Jones, A. Georges, A. Damascelli, A. J. Millis, Unveiling the underlying interactions in Ta_2NiSe_5 from photoinduced lifetime change. *Phys. Rev. B* **106**, L121106 (2022).

33. C. Lin, M. Ochi, R. Noguchi, K. Kuroda, M. Sakoda, A. Nomura, M. Tsubota, P. Zhang, C. Bareille, K. Kurokawa, Y. Arai, K. Kawaguchi, H. Tanaka, K. Yaji, A. Harasawa, M. Hashimoto, D. Lu, S. Shin, R. Arita, S. Tanda, T. Kondo, Visualization of the strain-induced topological phase transition in a quasi-one-dimensional superconductor $TaSe_3$. *Nat. Mater.* **20**, 1093–1099 (2021).

34. J. L. Cohn, B. D. White, C. A. M. Dos Santos, J. J. Neumeier, Giant Nernst effect and bipolarity in the quasi-one-dimensional metal $Li_{0.9}Mo_6O_{17}$. *Phys. Rev. Lett.* **108**, 056604 (2012).

35. J. Merino, R. H. McKenzie, Effective Hamiltonian for the electronic properties of the quasi-one-dimensional material $Li_{0.9}Mo_6O_{17}$. *Phys. Rev. B* **85**, 235128 (2012).

36. J. Z. Ke, C. Dong, H. P. Zhu, W. X. Liu, M. Y. Shi, Y. Q. Du, J. F. Wang, M. Yang, Synthesis and physical properties of the theoretically predicted spin-triplet superconductor $Li_{0.9}Mo_6O_{17}$. *Ceram. Int.* **47**, 25229–25235 (2021).

37. K. Dewhurst, S. Sharma, L. Nordström, F. Cricchio, F. Bultmark, O. Grånäs, E. K. U. Gross, The Elk FP-LAPW code (2025); http://elk.sourceforge.net.

38. J. P. Perdew, Y. Wang, Accurate and simple analytic representation of the electron-gas correlation energy. *Phys. Rev. B* **45**, 13244–13249 (1992).

39. J. P. Perdew, Y. Wang, Erratum: Accurate and simple analytic representation of the electron-gas correlation energy [Phys. Rev. B 45, 13244 (1992)]. *Phys. Rev. B* **98**, 079904 (2018).

40. J. Sebesta, O. Grånäs, Photo-induced manipulation and relaxation dynamics of Weyl-semimetals. *NPJ Comput. Mater.* **11**, 219 (2025).

41. M. F. Elhanoty, O. Eriksson, C. S. Ong, O. Grånäs, How quantum selection rules influence the magneto-optical effects of driven ultrafast magnetization dynamics. *Phys. Rev. B* **111**, 144410 (2025).

42. L. Dudy, J. W. Allen, J. D. Denlinger, J. He, M. Greenblatt, M. W. Haverkort, Y. Nohara, O. K. Andersen, Wannier orbital theory and angle-resolved photoemission spectroscopy for the quasi-one-dimensional conductor $LiMo_6O_{17}$. I. Six-band t2g Hamiltonian. *Phys. Rev. B* **109**, 115143 (2024).
